# Characteristics of suicide attempts in Northwestern Iran: a five-year population-based survey

**DOI:** 10.1186/s12888-023-05483-4

**Published:** 2024-01-02

**Authors:** Abbas Abbasi-Ghahramanloo, Mohammad Jafarzadeh, Farhad Pourfarzi, Sima Afrashteh, Ahad Azimi, Mustpha Ahmed Yusuf, Davoud Adham, Eslam Moradi-Asl

**Affiliations:** 1 Department of Public Health, School of Health, Ardabil University of Medical Sciences, Ardabil, Iran; 2 CDC of Ardabil Public Health, Ardabil University of Medical Sciences, Ardabil, Iran; 3 Department of Community Medicine, School of Medicine, Ardabil University of Medical Sciences, Ardabil, Iran; 4 Clinical Research Development Center, The Persian Gulf Martyrs Hospital, Bushehr University of Medical Sciences, Bushehr, Iran; 5 Department of Medical Microbiology and Parasitology, College of Health Sciences, Bayero University, Kano, Nigeria

**Keywords:** Suicide attempts, Subgrouping, Latent class analysis, Suicide trend, Iran

## Abstract

**Background:**

Suicide is a serious public health problem in the world. This study aims to describe the characteristics of suicide attempters in North-Western Iran and identify latent classes of suicide attempts.

**Methods:**

This cross-sectional study was conducted in Ardabil Province (Northwest Iran) during 2017–2021 based on a registration system for suicide attempts. We performed latent class analysis (LCA) using a procedure for LCA (PROC LCA) in SAS to investigate the subgroups of suicide attempters based on their characteristics and method, history, and outcome of suicide.

**Results:**

Three latent classes were identified for males and females; the first class (non-lethal attempters with lower educational levels) comprised 41.3% of males and 55.4% of females. The second class (non-lethal attempters with higher educational levels) described 52.4% of males and 42.7% of females. Finally, the third class (lethal attempters) included 6.4% of males and 1.9% of females. The main method of suicide attempts was poisoning with medications (87.3%). The results show that only 2.8% of people have a history of suicide attempts. Also, the suicide rate reached 8.26 per 100,000 population in 2021.

**Conclusion:**

The present study showed an increasing trend of suicide attempt incidence rate in Ardabil Province from 2017 (99.49 per 100,000 population) to 2021 (247.41 per 100,000 population). This means that the rate of change was 147.92 per 100,000 population during the study period. The findings of LCA, stress the necessity of identification and prioritization of unmet needs of people who had an incomplete suicide in Ardabil.

## Introduction

Suicide is a serious public health problem in the world [1]. According to the World Health Organization (WHO) statistics, more than 77% of suicides in 2022 occurred in low and income middle-income countries [2]. The incidence of suicide varies geographically. For example, South Korea has the highest suicide rate among the countries of the Organization for Economic Cooperation and Development (OECD) [2]. In Iran, although the suicide rate has decreased from 7.91 per 100,000 people to 5.2 per 100,000 people in recent years, suicide attempt is still very high (193.49 per 100,000 people) [3]. It seems that compared to Western countries, the suicide attempt rate in Iran is lower, but it is the highest rate among Middle Eastern countries [4]. Previous studies have identified several suicide risk factors [3, 5, 6]. For example, various factors in suicide attempts such as mental health problems, history of violence, high-risk sexual behaviors, drug abuse, attention-deficit hyperactivity disorder, and low socioeconomic have been reported in studies in different countries [3, 6, 7]. Evidence shows that one or more previous suicide attempts are the main predictor of death by suicide [8]. Identifying risk factors of suicide is possible with a sub-grouping of people who had an attempted suicide. LCA is a statistical modeling technique that allows heterogeneous individuals from a population to be grouped into smaller subgroups based on similar characteristics or behavioral patterns [9, 10]. Suicide is a costly event for the health systems, which is in contrast with the cultural values in Iran and it can hurt the survivors and their families. On the other hand, it is very beneficial for health policymakers to know the suicide incidence, trends, and factors for preventional planning [4]. The current study aims to describe the characteristics of suicide attempters in North-Western Iran and identify latent classes of suicide attempts.

## Methods

This cross-sectional study was conducted in Ardabil Province (Northwest Iran) from 2017 to 2021 based on a registration system for suicide attempts at Ardabil University of Medical Sciences (ARUMS). Participants were individuals who had attempted suicide during this study. More details of this registration system have been reported elsewhere [11]. LCA was used to investigate the subgroups of suicide attempters. To select the final model, a few indices were calculated and compared across seven models (Table 1). These indices were likelihood-ratio statistics G2, Akaike information criteria (AIC), Bayesian information criteria (BIC), entropy, and log-likelihood value. Besides, the interpretability and parsimony of a model could help in the selection of the final model [12]. The sex of participants was considered as a grouping variable. To perform simple statistical analysis, chi-square, Fisher’s exact test, and independent t-tests were used. SPSS version 16.0 was used for simple statistical analysis. LCA was performed using SAS version 9.4. In all analyses, the *P*-value of < 0.05 was considered statistically significant.

**Table 1:** Comparison of LCA Models with Different Latent Classes Based on Model Selection Statistics

Number of latent classes	Number of parameters estimated	G^2^	dF	AIC	BIC	Maximum log-likelihood
1	22	3,229.31	425	3,273.31	3,437.25	-31,954.91
2	46	1,351.68	401	1,443.68	1,786.44	-31,016.09
3	70	620.43	377	760.43	1,282.03	-30,650.46
4	94	446.32	353	634.32	1,334.76	-30,563.41
5	118	322.54	329	558.54	1,437.81	-30,501.52
6	142	257.28	305	541.28	1,599.39	-30,468.89
7	166	192.47	281	524.47	1,761.41	-30,436.48

## Results

**Fig. 1:**
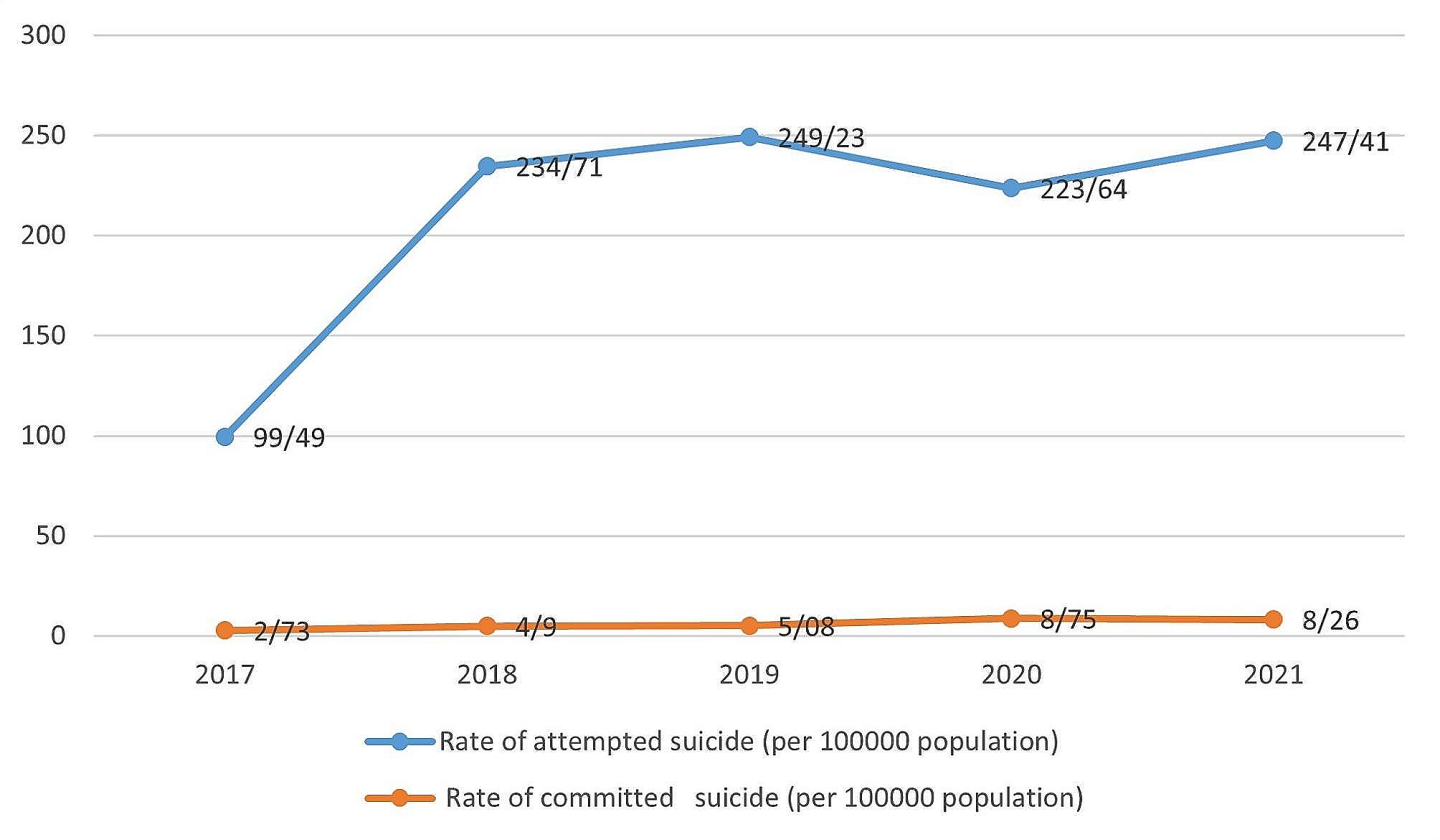
Trend of rate of attempted and committed suicide (per 100,000) citizens in Ardabil Province (2017–2021)

A total number of 12,734 records in the registration system for suicidal behaviors in Ardabil University of Medical Sciences were enrolled in this study. The main method of suicide attempt was poisoning with medications (87.3%). Table 2 represents the prevalence of using each method of suicide, the history of suicide attempts, as well as demographic characteristics. Figure 1 indicates the incidence and suicide rate from 2017 to 2021.

**Table 2:** Characteristics of the suicide attempters by outcome of suicide in Ardabil Province

Characteristics	Outcome of suicide		*P*-value	Total (n = 12,734)
	Incomplete	Complete		N (%)
	N (%)	N (%)		
**Age, Mean (SD)**	29.67(12.26)	36.06(16.14)	< 0.001	29.85(12.43)
**Gender**				
Male	5,753 (95.8)	251 (4.2)	< 0.001	6,004 (47.2)
Female	6,615 (98.4)	109 (1.6)		6,724 (52.8)
**Marital status**				
Single	6,731 (98.0)	138 (2.0)	< 0.001	6,869 (54.0)
Married	5,640 (96.2)	223 (3.8)		5,863 (46.0)
**Residency**				
Rural	1322 (94.9)	71(5.1)	< 0.001	1393 (11.0)
Urban	10,936 (97.5)	284 (2.5)		11,220 (89.0)
**Education**				
Illiterate	685 (95.7)	31(4.3)	< 0.001	716 (5.6)
Under diploma	4,195 (96.0)	176 (4.0)		4,371 (34.3)
Diploma	6,460 (98.3)	111 (1.7)		6,571 (51.6)
Academic	859 (96.3)	33(3.7)		892 (7.0)
**Method of suicide**				
Poisoning with substance	109 (94.8)	6(5.2)	< 0.001	115 (0.9)
Poisoning with poisons	436 (81.6)	98(18.4)		5,340 (4.2)
Poisoning with medications	10,978 (98.7)	141 (1.3)		11,119 (87.3)
Hanging	104 (51.2)	99(48.8)		203 (1.6)
Self-immolation	48(92.3)	4(7.7)		52(0.4)
Jumping from height	62(96.9)	2(3.1)		64(0.5)
Other ways	634 (98.3)	11(1.7)		645 (5.1)
**History of suicide**				
no	11,740 (98.0)	234 (2.0)	< 0.001	11,974 (94.0)
yes	246 (68.1)	115 (31.9)		361 (2.8)

Table 3 indicates the result of multiple logistic regression analysis of the relationship between the outcome of a suicide attempt and its related factors. The results showed that age (OR = 1.02, 95% CI: 1.01–1.03, *P*-value < 0.001), male gender (OR = 1.85, 95% CI: 1.41–2.45, *P*-value < 0.001), having a diploma and academic education (OR = 1.33, 95% CI: 1.02–1.75, *P*-value: 0.0036), and having a history of suicide attempt (OR = 20.02, 95% CI: 14.33–27.97, *P*-value < 0.001) was associated with the outcome of suicide. Moreover, poisoning with a substance (OR = 16.35, 95% CI: 8.35–32.04, *P*-value < 0.001), hanging (OR = 50.90, 95% CI: 25.21–102.80, *P*-value < 0.001), and self-immolation (OR = 7.85, 95% CI: 2.35–26.26, *P*-value: 0.001) increased the odds of death due to suicide attempt in comparison to jumping from height. Tables 4 and 5 show the results of the three-class model for male and female attempters respectively. Based on these findings, males and females were divided into three groups. The first class (non-lethal attempters with lower educational levels) comprised 41.3% of males and 55.4% of females. Also, the second class (non-lethal attempters with higher educational levels) described 52.4% of males and 42.7% of females. Finally, the third class (lethal attempters) included 6.4% of males and 1.9% of females. In both males and females, individuals in latent class 1 (non-lethal attempters with lower educational levels) were likely to have lower educational levels and be single and urban citizens. In this class, the probability of using poisoning with medications was high. In both males and females, those with second latent class membership were more likely to have a higher educational level, being married and urban citizens. Similar to the first class, in this class the probability of using poisoning with medications was high. Finally, in the third class, in both males and females, the probability of having a higher educational level, being married, and being urban citizens was high. Also, in this class the probability of having committed suicide was high.

**Table 3:** Multiple logistic regression analysis of the relationship between outcome of suicide and its related factors among suicide attempters in Ardabil Province

Characteristics	OR (95% CI)	*P* value
**Age**	1.02 (1.01–1.03)	< 0.001
**Sex (male/female)**	1.85 (1.41–2.45)	< 0.001
**Marital status (married/single)**	1.09 (0.81–1.48)	0.566
**Education (diploma and higher)**	1.33 (1.02–1.75)	0.036
**Method of suicide**		
Jumping from height	Ref	--
Poisoning with medications	2.48 (0.66–9.37)	0.181
Poisoning with substance	16.35 (8.35–32.04)	< 0.001
Poisoning with poisons	1.02 (0.54–1.94)	0.952
Hanging	50.90 (25.21–102.80)	< 0.001
Self-immolation	7.85 (2.35–26.26)	0.001
Other ways	2.89 (0.61–13.78)	0.183
**Having history of suicide**	20.02 (14.33–27.97)	< 0.001

**Table 4:** The three Latent Classes Model of suicide’s characteristics and risk factors among male Iranian subjects

	Latent class		
	Non-lethal attempters with lower educational level	Non-lethal attempters with higher educational level	Lethal attempters
**Latent class prevalence**	0.524	0.413	0.064
**Item-response probabilities**			
**Method of suicide**			
Poisoning with substance	0.000	0.036	0.022
Use of poisons	0.011	0.047	0.266
Poisoning with medications	**0.958**	**0.777**	0.339
Hanging	0.002	0.010	0.320
Self-immolation	0.000	0.012	0.003
Jumping from height	0.000	0.010	0.003
Other ways	0.028	0.110	0.046
**Education**			
Under diploma	**0.890**	0.269	0.399
Diploma and higher	0.109	**0.731**	**0.601**
**Marital status**			
Married	0.227	**0.608**	**0.603**
Single	**0.773**	0.392	0.397
**Residency**			
Rural	0.045	0.136	0.249
Urban	**0.955**	**0.864**	**0.751**
**Having history of suicide**	0.021	0.011	0.323
**Completed suicide**	0.003	0.000	**0.633**

*Note*. The probability of a “No” response can be calculated by subtracting the item-response probabilities shown above from 1

* Item-response probabilities > 0.5 in bold to facilitate interpretation

**Table 5:** The three Latent Classes Model of suicide’s characteristics and risk factors among female Iranian subjects

	Latent class		
	Non-lethal attempters with lower educational level	Non-lethal attempters with higher educational level	Lethal attempters
**Latent class prevalence**	0.554	0.427	0.019
**Item-response probabilities**			
**Method of suicide**			
Poisoning with substance	0.002	0.003	0.000
Use of poisons	0.001	0.083	0.304
Poisoning with medications	**0.981**	**0.814**	0.469
Hanging	0.000	0.008	0.172
Self-immolation	0.000	0.006	0.027
Jumping from height	0.005	0.007	0.009
Other ways	0.011	0.078	0.019
**Education**			
Under diploma	**0.742**	0.357	0.408
Diploma and higher	0.258	**0.643**	**0.592**
**Marital status**			
Married	0.336	**0.723**	**0.637**
Single	**0.664**	0.277	0.363
**Residency**			
Rural	0.015	0.259	0.214
Urban	**0.985**	**0.741**	**0.786**
**Having history of suicide**	0.006	0.034	0.281
**Completed suicide**	0.000	0.000	**0.862**

*Note*. The probability of a “No” response can be calculated by subtracting the item-response probabilities shown above from 1

* Item-response probabilities > 0.5 in bold to facilitate interpretation

## Discussion

The current study showed that the suicide attempts rate in 2021 was 247.41 and 8.26 per 100,000, which is relatively high compared to other regions of the country and the world [3, 13, 14]. The results of an analysis in 183 countries showed that an increase in lagged economic uncertainty, as well as in unemployment and economic growth, may lead to an increase in the risk of suicide [15]. Also, our results showed an increase in the general trend of suicide in the northwestern of Iran. The results of a national study in Iran showed that Iran has had the highest increase in suicide deaths among the countries of the Eastern Mediterranean Region (EMR) and Islamic countries. It is believed that national policies to prevent suicide have not been efficient enough and urgent intervention is needed [16]. According to the national prevention program in Iran, to prevent suicide, legal actions such as restricting access to common methods of suicide such as firearms or poisoning, reducing the stigma associated with suicide, treating mental disorders, especially depression, preventing these diseases, preventing drug abuse, involvement of and engagement with the media to improve the quality of reporting suicide attempts and to support and consultation people who have attempted suicide is necessary [16–18].

The results of our findings showed that the rate of suicide attempts is higher in women, while the chance of death is higher among men. Based on previous evidence, in general, death by suicide is more common among men than women [19]. Women are mostly suicidal gestures and do not intend to commit suicide, but in contrast to men, they have a more serious intention to die when attempting suicide [20]. Callanan et al. showed that men choose more violent methods to attempt suicide [21]. In addition, women are more willing to talk about their feelings, but men, despite suffering from symptoms such as anxiety, stress, or depression, never talk to anyone about their mental health due to traditional expectations around masculinity [22]. Göktas and et al. showed that the high rate of suicide in men may be due to alcohol abuse, the use of lethal methods and the tendency to violence compared to women. In addition, women are referred earlier and more often than men to receive health services, especially psychiatric services [23]. Also, men are more affected by violence in society and drug abuse [24, 25]. As a result, this reluctance to talk and the risk of substance abuse along with a drastic change in a person’s life can dramatically increase the risk of complete Suicide.

The findings of this study showed that having a history of suicide increases the odds of death by 20 times [26]. Evidence shows that the history of suicide is one of the most important predictors of complete suicide [27, 28]. Therefore, to prevent further actions leading to suicide, these people should not be left alone after being discharged from the hospital and should be under the supervision of mental health support institutions to receive the necessary intervention measures [26].

Using LCA, we identified three distinct groups of suicide attempts including non-lethal attempters with lower educational levels, non-lethal attempters with higher educational levels, and lethal attempters in both sexes. This finding shows that there are probably many unmet needs in the population of Ardabil Province, which has led them to commit suicide. These findings are consistent with a similar study in Iran [11]. Conducting exploratory studies and interviewing people who have failed to commit suicide can lead to identifying the main causes of suicide attempts.

LCA findings showed that higher educational levels have a high probability in two classes of non-lethal attempters with higher educational levels and lethal attempters. Also, based on logistic regression analysis, having a higher education increases the chance of death by suicide in the present study. People with higher education may be more at risk of suicide when facing life failures, public shame, and high premorbid functioning [29]. Urme et al. showed that students who committed suicide had a history of depression, despair, perfectionism, family conflicts, relationship break-ups, lack of social support, financial crisis, and academic stress before committing suicide [30]. Contrary to our findings, the results of other studies showed that people with higher educations are less likely to commit suicide [31, 32]. This finding in our study can be a wake-up call for higher education. Because if people with higher education become insecure about their job prospects and do not have hope for change and improvement of their conditions, the possibility of being pushed towards suicide increases among these persons [30].

The results of the present study showed that being married also has a high probability in the classes of non-lethal attempters with higher educational levels and lethal attempters in both sexes. Evidence shows a strong correlation between marital discord, marital dissatisfaction, and suicidal thoughts and attempts. Therefore, marital discord has an important relationship with the consequences of suicide and may be important in preventing this event [33]. In other countries, studies indicated that a status of being single is associated with a substantially increased risk of suicide in comparison with a status of being married [34, 35]. However, in a meta-analysis that studied marital status, no association was found between suicide ideation and suicide attempts with marital status [36]. An important issue in Iranian society is the social stigma of divorce and the insistence on continuing life, which causes frustration and family incompatibility, and as a result, can increase the probability of suicide [37]. These findings were in line with the present study.

Our findings revealed that being an urban resident had a high probability in all three latent classes among males and females. Increasing evidence suggests that the living place of individuals-particularly rural residents versus urban residents- may play an important role in their odds of dying by suicide [38–40]. Claire et al. concluded that rural adults do not differ significantly in terms of suicide distress behaviors compared to nonrural adults [41]. However, in Iran, the results are different. Mokhtari et al. Reported that most suicide attempters were urban residents [42] and Hajebi et al. revealed that being a rural resident is associated with lethal attempts [42]. Further studies are needed to the unique risk factors driving suicidality in rural and urban areas in Iran, as well as exploring heterogeneity in these factors across different countries.

The result of this study indicated that poisoning with medications was the most common means of suicide attempts. Drug poisoning was the main suicide method in Ilam and Hamadan provinces, according to studies conducted in Iran [34, 35]. In addition, a national study in Iran confirmed our findings that drug poisoning was the most common attempt suicide, while hanging was the most common suicide method [14]. Poisoning was also identified as the most common suicide method by studies in other countries, such as Poland and India [43, 44]. One of the most important reasons for the high prevalence of medication-related suicide attempts is the easy access of people to high-usage drugs such as acetaminophen, painkillers, antibiotics, and even benzodiazepines, and the lack of strictness of pharmacies in selling drugs without a doctor’s prescription in Iran. Also, people who use these drugs may think that they cannot cause severe harm because of ignorance. Therefore, their excessive consumption can be a good option for achieving suicide goals - including scaring people around and attracting attention [26].

In this study, the large sample size ensures that the results are in some measure representative of all suicide attempters Using the LCA approach as well, we were able to identify latent subgroups of suicide attempts. In addition, the present work had the following limitations: this study was delimited to the variables that were reported to ARUMS. Due to the cross-sectional nature of the study, no causal inferences were drawn. It was assumed that the collected data were accurate.

## Conclusion

The present study showed an increasing trend of suicide attempt incidence rate in Ardabil Province from 2017 (99.49 per 100,000 population) to 2021 (247.41 per 100,000 population). This means that the rate of change was 147.92 per 100,000 population during the study period. The findings of the present study stress the necessity of identification and prioritization of unmet needs of people who had an incomplete suicide in Ardabil.

## Data Availability

The datasets generated during and/or analyzed during the current study are not publicly available due to ethical reasons but are available from the corresponding author upon reasonable request.

## Abbreviations

WHO: World Health Organization
OECD: Organization for Economic Cooperation and Development
LCA: Latent Classes Analyses
